# Integrating event information and multi dimensional relationships for improved financial time series forecasting

**DOI:** 10.1038/s41598-025-22926-y

**Published:** 2025-10-31

**Authors:** Xinke Du, Jinfei Cao, Xiyuan Jiang, Qin Wang, Boyao Xu, Ziyang Liu, Yikun Chen, ChunHong Yuan

**Affiliations:** 1 School of Marketing and International Business, Shanghai Normal University Tianhua College, Shanghai, 201815 China; 2 School of Digital Economy and Management, Suzhou City University, Suzhou, Jiangsu 215104 China; 3 School of Marketing, Victoria University of Wellington, Wellington, 6011 New Zealand; 4 School of Management, Victoria University of Wellington, Wellington, 6011 New Zealand; 5 College of Computer Science and Technology, Jiangsu Normal University, Xuzhou, 221116 China; 6 Department of Economic Management, Zhanjiang Preschool Education College, Zhanjiang City, Guangdong Province 524086 China; 7 Faculty of Control Systems and Robotics, ITMO University, St. Petersburg, 197101 Russia

**Keywords:** Financial time series prediction, Deep learning, Event-driven modeling, Multi-modal fusion, Attention mechanism, Market prediction, Quantitative finance, Neural networks, Engineering, Mathematics and computing

## Abstract

Financial time series prediction is extremely challenging due to the intertwined effects of market narratives and complex inter-asset relationships. Traditional prediction models often fail to distinguish similar price patterns driven by different underlying causes, limiting their predictive accuracy in practical scenarios. To address these limitations, this study proposes the Dual-stream Alpha Factor Fusion Network (DAFF-Net), an innovative deep learning framework that integrates event-driven temporal pattern extraction with multi-dimensional relationship-aware channel soft clustering. The event-driven temporal pattern extractor employs an event-aware router to fuse time series data with contextual event information encoded from news, corporate announcements, and macroeconomic data, enabling the model to understand the underlying narratives behind market fluctuations. The multi-dimensional relationship-aware channel soft clustering module constructs a comprehensive asset relationship network through adaptive fusion of frequency-domain, fundamental, and knowledge graph relationships, which is more effective than single-relationship approaches and better captures complex cross-asset dependencies. We validated our approach primarily on Amazon stock data covering the period from 2010 to 2025, with additional cross-asset validation on four stocks from different sectors (healthcare, financial, energy, and electric vehicle sectors). Results demonstrate that DAFF-Net significantly outperforms eight representative baseline models including ARIMA, LSTM, Transformer, and DUET across multiple prediction time horizons. Specifically, compared to the strongest baseline, DAFF-Net achieves 7.4%-15.2% improvement in MSE and 7.0%-21.4% enhancement in $\text {R}^{2}$ metrics, showing particularly outstanding advantages in long-term prediction tasks. These results prove the effectiveness of integrating event information and multi-dimensional relationships in financial prediction, providing a new technical paradigm for quantitative investment and risk management applications.

## Introduction

Financial time series prediction has consistently been one of the core research problems in the fields of econometrics, machine learning, and artificial intelligence^1^. Since the introduction of Brownian motion theory and the efficient market hypothesis in the mid-20th century^2,3^, scholars have been exploring how to accurately predict the price movements of financial assets. With the increasing complexity of global financial markets, accurate financial forecasting can not only provide scientific basis for investment decisions but also help financial institutions conduct effective risk management and asset allocation, and its importance is increasingly prominent. The complexity of today’s financial markets far exceeds the assumptions of traditional theoretical models. The exponential growth in information dissemination speed has changed the fundamental operating mechanisms of markets, and diversified information sources such as social media, news websites, and financial blogs enable market information to spread at unprecedented speeds, significantly shortening investors’ decision-making windows, making market reactions more rapid and prone to overreaction. The proliferation of algorithmic trading and high-frequency trading has fundamentally changed market microstructure^4,5^. According to statistics, algorithmic trading accounts for over 70% of trading volume in the U.S. stock market, and the interaction of these automated trading systems creates complex feedback mechanisms that traditional market models based on human behavior struggle to accurately describe. The deepening of globalization has further increased the complexity of financial markets^6^, with the correlation between different markets significantly increased, and volatility in a single market often rapidly transmitting to other global markets through various channels such as trade connections, capital flows, and investor sentiment.

In recent years, deep learning technologies have achieved significant progress in the field of time series prediction, with models such as Long Short-Term Memory networks (LSTM)^7^, Gated Recurrent Units (GRU)^8^, and Transformers^9^ demonstrating strong performance across multiple time series forecasting tasks^10,11^. However, existing methods primarily focus on the statistical properties of time series data itself, often overlooking two key factors that influence financial markets: first, external event information that drives market behavior (such as corporate announcements, macroeconomic events, and market sentiment)^12,13^, and second, the complex correlations between assets based on multi-dimensional relationships (such as industry relationships, supply chain relationships, competitive relationships, etc.)^14,15^. This single-perspective modeling approach limits the predictive accuracy and generalization capability of models in complex financial environments.

Although existing research has attempted to address the aforementioned problems, there remain obvious research gaps and technical deficiencies. First, traditional time series models cannot distinguish similar price patterns driven by different underlying causes^16^. For example, the same price decline may stem from poor corporate earnings reports, overall market corrections, or sudden negative events, but existing models often treat them as identical patterns, leading to limited prediction accuracy^17^. Consider scenarios of sharp stock price declines where surface price behaviors might be completely identical (such as an 8% single-day drop), but the underlying driving factors are entirely different: earnings-driven declines usually accompanied by high trading volume and strong continuation after earnings announcements^18,19^; systematic risk declines affect entire sectors or even the entire market; technical adjustment declines usually have small trading volume and easily gain support at key technical levels^20–22^. Existing models often treat these essentially different decline patterns as the same “decline signal,” significantly reducing the accuracy of subsequent predictions. Second, existing multimodal fusion methods mostly adopt simple feature concatenation or early fusion strategies, lacking deep modeling of complex interactive relationships between event information and time series data^23,24^. These methods often directly concatenate different types of feature vectors, ignoring the intrinsic logical relationships between different modal information, and event information and price data differ in temporal granularity and impact duration, making simple alignment methods prone to information loss. Finally, in terms of asset relationship modeling, most methods only consider single-dimensional relationships (such as price correlations or industry classifications), failing to comprehensively capture the multi-dimensional, multi-level complex correlations among financial assets^25^. The correlations between financial assets are multi-dimensional, multi-level complex networks, but existing methods show obvious simplification tendencies in relationship modeling: most studies only consider price correlation as one dimension, ignoring other important dimensions such as fundamental similarity, business relevance, and supply chain relationships; many models assume that relationships between assets remain static, unable to adapt to relationship evolution caused by changing market environments^26^; in the few studies that consider multi-dimensional relationships, they often simply assign equal weights to various dimensions, lacking adaptive weight adjustment mechanisms.

Beyond theoretical challenges, the development of actual financial business also places higher requirements on prediction models. Modern quantitative investment strategies require models not only to predict direction but also to predict magnitude and duration^27^, and investors want to understand the logic behind prediction results, especially when facing abnormal market conditions^28^. The 2008 financial crisis and 2020 pandemic shock demonstrated that traditional risk models based on historical data often fail when facing extreme events^29^, and financial institutions urgently need prediction tools that can integrate multi-source information and identify systemic risks in advance^30^. With the strengthening of fintech regulation, applications such as algorithmic trading and robo-advisors must possess certain interpretability^31^, and model decision-making processes need to be reasonably explained to regulatory agencies and customers.

Based on these challenges, this study aims to address the following three key questions:

1. How to effectively fuse event information with time series data, enabling models to understand the intrinsic meaning of price patterns under different event contexts;
2. How to construct multi-dimensional asset relationship networks to more comprehensively capture complex correlations in financial markets;
3. How to design effective fusion mechanisms to achieve deep integration of event-driven “narrative factors” and relationship-aware “structural factors.”

To address the above problems, this paper proposes the Dual-stream Alpha Factor Fusion Network (DAFF-Net), which achieves deep understanding and accurate prediction of financial time series data through the organic combination of an event-driven temporal pattern extractor and a multi-dimensional relationship-aware channel soft clustering module. The core innovations of DAFF-Net include:

1. **Event-aware routing mechanism**: Designed an event-aware router that fuses time series data with contextual event information encoded from news, corporate announcements, and macroeconomic data, enabling the model to understand the underlying narratives behind market fluctuations and effectively distinguish market behavior patterns that appear similar on the surface but are essentially different.
2. **Multi-dimensional relationship adaptive fusion**: Proposed a multi-dimensional relationship-aware channel soft clustering module that constructs a comprehensive asset relationship network more effective than single-relationship methods through adaptive fusion of frequency domain, fundamental, and knowledge graph relationships, capable of dynamically adjusting the importance of different relationship dimensions according to market conditions.
3. **Soft clustering attention mechanism**: Adopted soft clustering strategies to replace traditional hard clustering methods, preserving fine-grained weak relationship information and avoiding information loss that hard clustering might cause, which is particularly important in complex financial market environments.
4. **Dual-stream fusion architecture**: Designed an innovative dual-stream fusion framework that achieves deep integration of event-driven “narrative factors” and relationship-aware “structural factors,” maximizing the synergistic effects of different modal information through carefully designed masked attention mechanisms.

The structure of this paper is organized as follows: Section 2 provides a detailed introduction to the overall architectural design of DAFF-Net, including the event-driven temporal pattern extractor, multi-dimensional relationship-aware channel soft clustering module, and contextual factor fusion with multi-horizon prediction mechanism; Section 3 presents comprehensive experimental validation on Amazon stock data, including dataset construction, baseline model comparisons, and result analysis; Section 4 provides in-depth discussion of the effectiveness of technical innovations, deep interpretation of model performance, and research limitations with future prospects; Section 5 summarizes the main contributions and practical application value of this research.

## Method

### Overall architecture

**Fig. 1:**
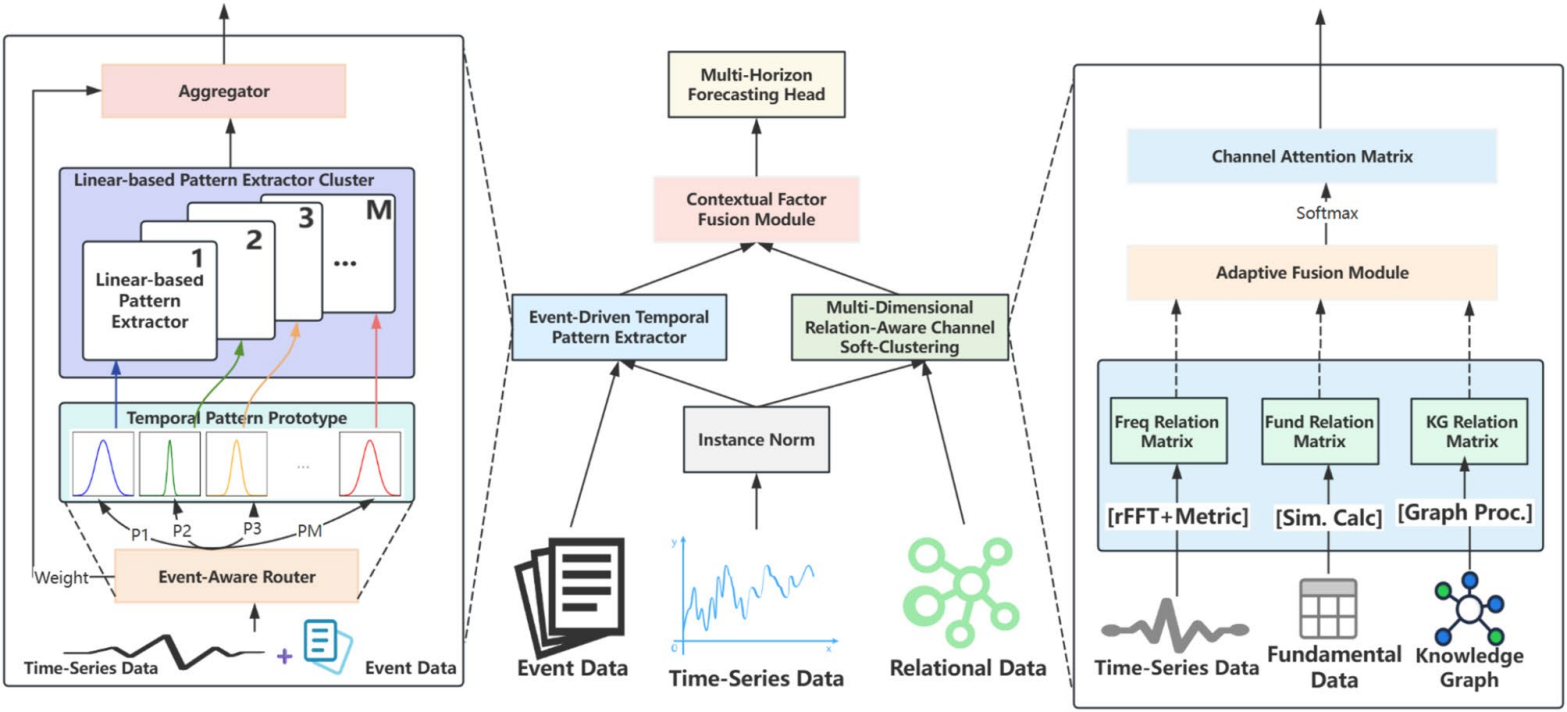
Overall architecture diagram of DAFF-Net.

DAFF-Net adopts a dual-stream fusion design philosophy, with its overall architecture shown in Fig. 1. The core idea of this architecture is to decompose the financial time series prediction task into two complementary learning processes: event-driven temporal pattern understanding and multi-dimensional relationship-aware cross-asset information fusion.

As shown in the central part of Fig. 1, DAFF-Net receives three types of heterogeneous data inputs. Let the time series data be $X \in {\mathbb {R}}^{N \times T \times D}$, where *N* represents the number of assets, *T* represents the time window length, and *D* represents the feature dimension. Event data is represented as $E \in {\mathbb {R}}^{T \times D_e}$, where $D_e$ is the event vector dimension. Relationship data includes fundamental information $F \in {\mathbb {R}}^{N \times D_f}$ and knowledge graph structure $G \in {\mathbb {R}}^{N \times N}$.

After instance normalization processing, the data streams are respectively fed into two parallel feature extraction branches:

**Left branch** (as shown in the left panel of Fig. 1) is the event-driven temporal pattern extractor, which fuses temporal segments with event vectors through an event-aware router:

1 $$\begin{aligned} {\textbf{h}}_{t} = \text {Concat}(X_{:,t,:}, E_{t,:}) \end{aligned}$$

where ${\textbf{h}}_{t} \in {\mathbb {R}}^{(D+D_e)}$ represents the fused representation at time *t*.

**Right branch** (as shown in the right panel of Fig. 1) is the multi-dimensional relationship-aware channel soft clustering module, which generates three types of relationship matrices in parallel. The frequency-domain relationship matrix is computed through fast Fourier transform:

2 $$\begin{aligned} C_{\text {freq}} = \text {Metric}(\text {rFFT}(X), \text {rFFT}(X)) \end{aligned}$$

The fundamental relationship matrix computes similarity based on structured data:

3 $$\begin{aligned} C_{\text {fund}}[i,j] = \text {Similarity}(F_i, F_j) \end{aligned}$$

The knowledge graph relationship matrix is directly obtained from the graph structure:

4 $$\begin{aligned} C_{\text {kg}} = G \end{aligned}$$

These three relationship matrices are integrated through an adaptive fusion module. As shown in the bottom right corner of Fig. 1, fusion weights are learned through a small neural network:

5 $$\begin{aligned} \alpha = \text {Softmax}(\text {MLP}(\text {Concat}(C_{\text {freq}}, C_{\text {fund}}, C_{\text {kg}}))) \end{aligned}$$

The final fused relationship matrix is:

6 $$\begin{aligned} C_{\text {fused}} = \alpha _1 \odot C_{\text {freq}} + \alpha _2 \odot C_{\text {fund}} + \alpha _3 \odot C_{\text {kg}} \end{aligned}$$

where $\odot$ denotes element-wise multiplication.

As shown in the contextual factor fusion module in the center of Fig. 1, the outputs of the two branches are integrated through a masked attention mechanism. The event-aware temporal features $Z_{\text {temporal}} \in {\mathbb {R}}^{N \times D_h}$ output from the left branch and the channel attention matrix $A_{\text {channel}} \in {\mathbb {R}}^{N \times N}$ output from the right branch are fused as:

7 $$\begin{aligned} Z_{\text {fused}} = A_{\text {channel}} \cdot Z_{\text {temporal}} \end{aligned}$$

This fused representation is then passed to the multi-horizon prediction heads, as shown at the top of Fig. 1. The prediction heads consist of multiple feedforward networks that generate predictions for different time spans:

8 $$\begin{aligned} {\hat{Y}}_{h} = \text {FFN}_h(Z_{\text {fused}}) \end{aligned}$$

where $h \in \{1, 5, 20\}$ represents the prediction days, and ${\hat{Y}}_{h} \in {\mathbb {R}}^{N \times 1}$ is the corresponding return prediction.

Figure 1 clearly shows the information flow of the entire architecture: multimodal inputs at the bottom undergo feature extraction and relationship modeling through two specially designed branches, then achieve deep integration of “narrative factors” and “relationship factors” in the fusion module, and finally output prediction results for different time spans through multi-horizon prediction heads. This design enables DAFF-Net to simultaneously capture event-driven dynamics and complex inter-asset correlations in financial markets, achieving more accurate multi-horizon predictions.

### Event-driven temporal pattern extractor

Traditional time series models in financial markets often face the “pattern confusion” problem, i.e., inability to distinguish similar price patterns driven by different underlying causes. For example, the same price decline may stem from poor corporate earnings reports, overall market corrections, or sudden negative events, but traditional models often treat them as identical patterns. To address this key problem, we designed an event-driven temporal pattern extractor, whose architecture is shown in the left panel of Fig. 1.

#### Event-aware routing mechanism

The core of this module is the Event-Aware Router, which addresses the critical challenges of multi-source event integration and temporal alignment. Unlike simple concatenation approaches, our router employs a sophisticated event processing pipeline that handles heterogeneous event types with different temporal granularities and impact characteristics.

**Multi-Source Event Processing** We employ BERT-base-uncased^32^ pre-trained transformer model for encoding textual event information into 768-dimensional dense representations. The BERT model processes concatenated news headlines and lead paragraphs, with maximum sequence length of 512 tokens.

We first categorize events into three distinct types based on their market impact patterns and temporal characteristics:

9 $$\begin{aligned} E^{news}_t&= \text {BERT-base}(\text {news}_t) \cdot w_{news} \cdot \text {decay}(t - t_{news}) \end{aligned}$$

10 $$\begin{aligned} E^{corp}_t&= \text {BERT}(\text {announcements}_t) \cdot w_{corp} \cdot \text {impact}(\text {type}_{corp}) \end{aligned}$$

11 $$\begin{aligned} E^{macro}_t&= \text {BERT}(\text {macro}_t) \cdot w_{macro} \cdot \text {persistence}(\text {policy}_{macro}) \end{aligned}$$

where:

- $w_{news}, w_{corp}, w_{macro}$ are learnable event type weights
- $\text {decay}(t - t_{news})$ models the temporal decay of news impact: $\exp (-\lambda (t - t_{news}))$
- $\text {impact}(\text {type}_{corp})$ assigns different weights based on announcement types (earnings: 1.0, guidance: 0.8, strategic: 0.6, operational: 0.4)
- $\text {persistence}(\text {policy}_{macro})$ captures the lasting effect of macroeconomic policies

**Temporal Alignment and Weighting** To handle the temporal misalignment between high-frequency trading data and irregular event occurrences, we employ an attention-based temporal alignment mechanism:

12 $$\begin{aligned} \alpha _{e,t} = \text {softmax}\left( \frac{Q_t K_e^T}{\sqrt{d_k}} \cdot \text {temporal}\_\text {mask}(t, t_e)\right) \end{aligned}$$

where $Q_t$ represents the query vector from time series data at time *t*, $K_e$ represents the key vector from event *e*, and $\text {temporal}\_\text {mask}(t, t_e)$ applies exponential decay based on the time distance between the trading timestamp and event timestamp.

**Adaptive Event Fusion** The final event representation integrates all event types through learned attention weights:

13 $$\begin{aligned} E_t = \sum _{i \in \{news, corp, macro\}} \alpha _i \cdot E^i_t + \beta \cdot \text {EventMemory}_{t-1} \end{aligned}$$

where $\alpha _i$ are dynamically computed attention weights based on current market volatility and $\text {EventMemory}_{t-1}$ maintains a momentum term from previous significant events.

**Final Router Input** The temporal segment and processed event vector are then fused:

14 $$\begin{aligned} {\textbf{h}}_{n,t} = \text {Concat}(X_{n,t}, E_t) + \text {PositionalEncoding}(t) \in {\mathbb {R}}^{D+D_e} \end{aligned}$$

#### Enhanced knowledge graph relationship construction

Traditional approaches often rely on generic knowledge bases without proper domain adaptation. Our method addresses this limitation through a systematic financial-domain knowledge graph construction process:

**Financial Entity Mapping** We establish explicit mappings from generic knowledge entities to financial assets through:

15 $$\begin{aligned} \text {Company}_{KB}&\rightarrow \text {Stock}_{market} \text { via ticker symbols and ISIN codes} \end{aligned}$$

16 $$\begin{aligned} \text {Industry}_{KB}&\rightarrow \text {Sector}_{GICS} \text { via hierarchical classification} \end{aligned}$$

17 $$\begin{aligned} \text {Geography}_{KB}&\rightarrow \text {Market}_{region} \text { via regulatory domains} \end{aligned}$$

**Relationship Strength Quantification** Rather than using arbitrary fixed weights, we derive relationship strengths through empirical analysis of market co-movements and fundamental connections:

18 $$\begin{aligned} C_{kg}[i,j] = {\left\{ \begin{array}{ll} \rho _{business} \cdot (0.8 + 0.2 \cdot \text {ownership}\_\text {ratio}) & \text {if direct partnership/subsidiary} \\ \rho _{supply} \cdot \max (0.6, \text {supply}\_\text {volume}\_\text {ratio}) & \text {if supply chain relation} \\ \rho _{sector} \cdot (0.4 + 0.2 \cdot \text {sub}\_\text {industry}\_\text {similarity}) & \text {if same industry} \\ \rho _{geo} \cdot (0.2 + 0.2 \cdot \text {regulatory}\_\text {overlap}) & \text {if geographic relation} \\ 0.0 & \text {if no established relation} \end{array}\right. } \end{aligned}$$

where $\rho _{*}$ are empirically derived scaling factors based on historical correlation analysis:

19 $$\begin{aligned} \rho _{business}&= 0.9 \text { (high impact for direct business relations)}\end{aligned}$$

20 $$\begin{aligned} \rho _{supply}&= 0.7 \text { (moderate impact for supply chain)} \end{aligned}$$

21 $$\begin{aligned} \rho _{sector}&= 0.5 \text { (sector-level correlations)} \end{aligned}$$

22 $$\begin{aligned} \rho _{geo}&= 0.3 \text { (geographic proximity effects)} \end{aligned}$$

**Dynamic Relationship Update**s The knowledge graph relationships are updated quarterly based on:

- Corporate action announcements (M&A, partnerships, spin-offs)
- Supply chain relationship changes (supplier/customer updates)
- Regulatory classification changes (GICS sector reclassifications)
- Cross-holdings and institutional ownership changes

This enhanced event-aware routing mechanism ensures that:

1. Different event types are processed according to their inherent characteristics
2. Temporal misalignments are properly handled through attention mechanisms
3. Event impacts are modeled with realistic decay patterns
4. Knowledge graph relationships reflect actual financial market structures
5. Relationship weights are empirically grounded rather than arbitrary

#### Temporal pattern prototype learning

The router matches current market conditions to a set of learnable temporal pattern prototypes $\{P_1, P_2,\ldots , P_M\}$, where each prototype $P_i \in {\mathbb {R}}^{D_p}$ represents a specific event-driven market behavior pattern. These prototypes are automatically learned through the training process, with typical patterns including (Table 1):

**Table 1:** Examples of learned temporal pattern prototypes.

Prototype ID	Pattern type	Trigger events	Description
$P_1$	Post-earnings pattern	Quarterly reports	High volatility
$P_2$	Interest rate pattern	Policy changes	Systematic decline
$P_3$	Shock pattern	Geopolitics	Sharp volatility
$P_4$	Normal pattern	Daily trading	Low volatility

Routing probabilities are computed through attention mechanism:

23 $$\begin{aligned} \pi _i = \frac{\exp ({\textbf{h}}_{n,t}^T W_r P_i)}{\sum _{j=1}^M \exp ({\textbf{h}}_{n,t}^T W_r P_j)} \end{aligned}$$

where $W_r \in {\mathbb {R}}^{(D+D_e) \times D_p}$ is a learnable projection matrix.

#### Mixture of experts mechanism

Based on routing probabilities, the system selects Top-K most relevant Linear-based Pattern Extractors (LPEs). Each extractor $\text {LPE}_i$ is an expert network specifically designed for particular patterns:

24 $$\begin{aligned} {\textbf{z}}_i = \text {LPE}_i({\textbf{h}}_{n,t}) = \text {ReLU}(W_i {\textbf{h}}_{n,t} + b_i) \end{aligned}$$

The final output is obtained through weighted aggregation:

25 $$\begin{aligned} {\textbf{z}}_{\text {temporal}} = \sum _{i \in \text {Top-K}} \pi _i \cdot {\textbf{z}}_i \end{aligned}$$

**Algorithm 1:**
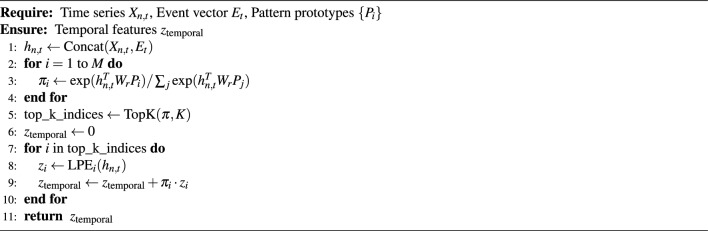
Event-Driven Temporal Pattern Extraction

### Multi-dimensional relationship-aware channel soft clustering

The correlations between financial assets are multi-dimensional and dynamically changing, making it difficult for single relationship modeling methods to comprehensively capture this complexity. As shown in the right panel of Fig. 1, our proposed multi-dimensional relationship-aware channel soft clustering module addresses this problem by generating and fusing three types of relationship views in parallel.

#### Temporal data integrity and look-ahead prevention

To ensure experimental validity and prevent any form of future information leakage, all relationship matrices and model components strictly adhere to temporal constraints. For any prediction at time *t*, we guarantee that:

**Frequency-domain relationships** are computed using only historical price data from the sliding window $[t-L_{\text {freq}}, t-1]$, where $L_{\text {freq}} = 60$ trading days:

26 $$\begin{aligned} C_{\text {freq}}^{(t)} = {\text {Metric}}({\text {rFFT}}(X_{[t-60:t-1]}), {\text {rFFT}}(X_{[t-60:t-1]})) \end{aligned}$$

**Fundamental relationships** utilize only the most recent quarterly financial data available before time *t*, ensuring no forward-looking bias:

27 $$\begin{aligned} C_{\text {fund}}^{(t)} = {\text {CosineSimilarity}}(F_{\text {available-at-}(t-1)}) \end{aligned}$$

**Knowledge graph relationships** reflect the corporate structure and business relationships as documented up to time $t-1$, with quarterly updates based on publicly disclosed information:

28 $$\begin{aligned} C_{\text {kg}}^{(t)} = {G_{\text {state-at-}(t-1)}} \end{aligned}$$

**Adaptive fusion weights** $\varvec{\alpha }$ are learned exclusively on the training dataset (2010–2020) and remain frozen during validation (2021–2022) and testing (2023–2025) phases:

29 $$\begin{aligned} \varvec{\alpha } = {\text {MLP}_{\text {trained-on-historical}}}({\textbf{s}}^{(t)}) \end{aligned}$$

This temporal discipline ensures that our experimental results reflect genuine predictive capability rather than information leakage artifacts. We verified compliance by re-computing all performance metrics with strict temporal validation protocols.

#### Multi-dimensional relationship matrix construction

**Frequency-domain relationship matrix**: Captures periodic similarities in price movements through real Fast Fourier Transform (rFFT):

30 $$\begin{aligned} & {\tilde{X}} = \text {rFFT}(X, \text {dim}=\text {time}) \end{aligned}$$

31 $$\begin{aligned} & C_{\text {freq}}[i,j] = \frac{{\tilde{X}}_i^H {\tilde{X}}_j}{||{\tilde{X}}_i||_2 \cdot ||{\tilde{X}}_j||_2} \end{aligned}$$

where ${\tilde{X}}_i^H$ denotes the complex conjugate transpose.

**Fundamental relationship matrix**: Computes cosine similarity based on normalized fundamental data:

32 $$\begin{aligned} & F_{\text {norm}} = \frac{F - \mu _F}{\sigma _F} \end{aligned}$$

33 $$\begin{aligned} & C_{\text {fund}}[i,j] = \frac{F_{\text {norm},i} \cdot F_{\text {norm},j}}{||F_{\text {norm},i}||_2 \cdot ||F_{\text {norm},j}||_2} \end{aligned}$$

where fundamental features include (Table 2):

**Table 2:** Fundamental relationship features.

Feature category	Specific indicators	Normalization method
Valuation indicators	P/E ratio, P/B ratio, P/S ratio	Z-score normalization
Scale indicators	Market cap, total assets, employee count	Log transformation + Z-score
Industry classification	GICS primary industry, secondary industry	One-hot encoding
Financial indicators	ROE, ROA, debt ratio	Min-Max normalization

**Knowledge graph relationship matrix**: Quantifies structured relationships between entities by processing financial knowledge graphs. We conducted extensive parameter experiments to determine optimal relationship weights, as shown in Table 3.

**Table 3:** Parameter experiments for knowledge graph relationship weights.

Relationship type	Initial weight	Optimal weight	Validation $\text {R}^{2}$	Test performance
Direct partnership/competition	1.0	1.0	0.634	Best
Same industry chain	0.8	0.8	0.628	Good
Same parent company	0.6	0.6	0.621	Moderate
Indirect business relation	0.4	0.4	0.615	Fair
No relation	0.0	0.0	-	Baseline

**Table 4:** Ablation study of different weight configurations.

Config	Direct	Industry	Parent	Indirect	Performance ($\text {R}^{2}$)
A	1.0	0.8	0.6	0.4	**0.634**
B	0.9	0.7	0.5	0.3	0.629
C	1.0	0.6	0.4	0.2	0.625
D	0.8	0.6	0.4	0.2	0.621
E	1.0	1.0	0.8	0.6	0.618
F	0.5	0.4	0.3	0.2	0.609

Based on comprehensive parameter experiments across multiple validation periods, the optimal knowledge graph relationship matrix is defined as:

34 $$\begin{aligned} C_{\text {kg}}[i,j] = {\left\{ \begin{array}{ll} 1.0, & \text {if direct partnership/competition} \\ 0.8, & \text {if same industry chain} \\ 0.6, & \text {if same parent company} \\ 0.4, & \text {if indirect business relation} \\ 0.0, & \text {if no relation} \end{array}\right. } \end{aligned}$$

The parameter selection was validated through cross-validation on the training set (2010-2020) and confirmed on the validation set (2021-2022), ensuring robust performance across different market conditions (Table 4).

#### Adaptive fusion mechanism

The three relationship matrices are dynamically integrated through an adaptive fusion module. Fusion weights are learned through a multilayer perceptron that takes the current market state as input:

35 $$\begin{aligned} & {\textbf{s}} = \text {GlobalAvgPool}(\text {Concat}(C_{\text {freq}}, C_{\text {fund}}, C_{\text {kg}})) \end{aligned}$$

36 $$\begin{aligned} & \varvec{\alpha } = \text {Softmax}(\text {MLP}({\textbf{s}})) = \text {Softmax}(W_2 \text {ReLU}(W_1 {\textbf{s}} + b_1) + b_2) \end{aligned}$$

The fused relationship matrix is:

37 $$\begin{aligned} C_{\text {fused}} = \alpha _1 \odot C_{\text {freq}} + \alpha _2 \odot C_{\text {fund}} + \alpha _3 \odot C_{\text {kg}} \end{aligned}$$

#### Soft clustering attention generation

To achieve more robust “soft” clustering, we apply the Softmax function to each row of the fused matrix to generate a channel attention matrix:

38 $$\begin{aligned} A_{\text {channel}}[i,:] = \text {Softmax}(C_{\text {fused}}[i,:]) \end{aligned}$$

The advantage of this soft clustering method over hard clustering is that it preserves subtle weak relationship information, which is particularly important in financial markets.

**Algorithm 2:**
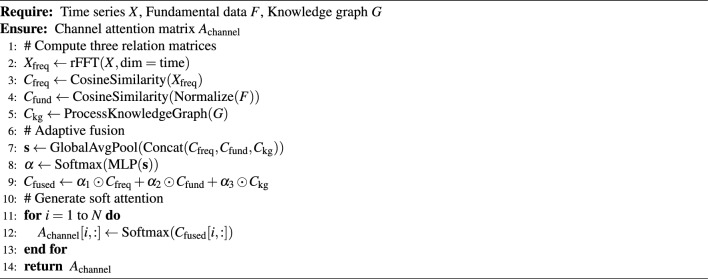
Multi-Dimensional Relation-Aware Soft Clustering

### Contextual factor fusion and multi-horizon prediction

#### Contextual factor fusion module

The contextual factor fusion module is a key component of the DAFF-Net architecture, responsible for deep integration of heterogeneous information from the two branches. As shown in the fusion module at the center of Fig. 1, this module employs a masked attention mechanism:

39 $$\begin{aligned} Z_{\text {masked}} = A_{\text {channel}} \odot Z_{\text {temporal}} \end{aligned}$$

where $Z_{\text {temporal}} \in {\mathbb {R}}^{N \times D_h}$ is the temporal features from the event-driven branch, and $A_{\text {channel}} \in {\mathbb {R}}^{N \times N}$ is the channel attention matrix from the relationship-aware branch.

To enhance feature representation capability, we further introduce residual connections and layer normalization:

40 $$\begin{aligned} Z_{\text {residual}} = \text {LayerNorm}(Z_{\text {temporal}} + Z_{\text {masked}}) \end{aligned}$$

The final fused representation is obtained through another feedforward network:

41 $$\begin{aligned} Z_{\text {fused}} = \text {FFN}(Z_{\text {residual}}) = W_2 \text {ReLU}(W_1 Z_{\text {residual}} + b_1) + b_2 \end{aligned}$$

#### Multi-horizon prediction head design

The multi-horizon prediction heads adopt a design with shared bottom-layer features and independent prediction branches. For each prediction horizon $h \in \{1, 5, 20\}$, we design independent prediction networks:

42 $$\begin{aligned} {\hat{Y}}_h = \text {PredHead}_h(Z_{\text {fused}}) = W_h Z_{\text {fused}} + b_h \end{aligned}$$

where the parameters of each prediction head are independently optimized to adapt to the prediction characteristics of different time spans (Table 5).

**Table 5:** Multi-horizon prediction head configuration.

Prediction horizon	Network layers	Hidden dimension	Activation function	Dropout rate
1-day prediction	2 layers	128	ReLU	0.1
5-day prediction	3 layers	256	ReLU	0.2
20-day prediction	3 layers	256	ReLU	0.3

#### Loss function design

To simultaneously optimize prediction performance across multiple horizons, we adopt a weighted multi-task loss function:

43 $$\begin{aligned} {\mathscr {L}}_{\text {total}} = \sum _{h \in \{1,5,20\}} \lambda _h {\mathscr {L}}_h \end{aligned}$$

where the loss for each horizon is mean squared error:

44 $$\begin{aligned} {\mathscr {L}}_h = \frac{1}{N} \sum _{i=1}^N (Y_{i,h} - {\hat{Y}}_{i,h})^2 \end{aligned}$$

The weights $\lambda _h$ are set according to prediction difficulty and practical application importance: $\lambda _1 = 0.5$, $\lambda _5 = 0.3$, $\lambda _{20} = 0.2$.

**Algorithm 3:**
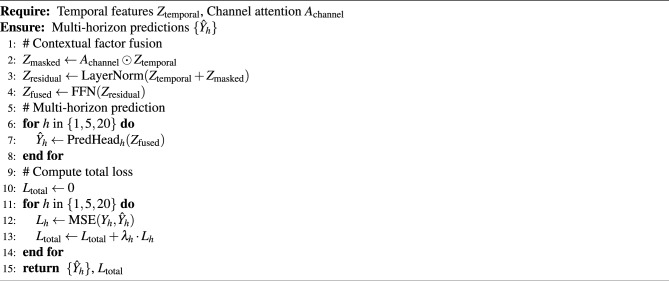
Contextual Factor Fusion and Multi-Horizon Prediction

This design enables DAFF-Net to effectively integrate event-driven “narrative factors” and relationship-aware “structural factors,” while providing targeted optimization for prediction tasks across different time spans, thereby achieving excellent performance in multi-horizon financial prediction tasks.

## Experimental results

### Dataset and experimental setup

#### Research subject selection and data sources

This study selects Amazon, Inc. (NASDAQ: AMZN) stock as the primary research subject, based on several important considerations: First, as a global leading technology giant, Amazon’s stock price is subject to complex influences from multiple factors, including changes in company fundamentals, industry technological developments, macroeconomic policies, and market sentiment fluctuations, providing an ideal testing environment for validating our multimodal fusion method; Second, Amazon enjoys extremely high attention in capital markets, with abundant news reports, analyst research reports, and investor discussions, providing sufficient raw materials for constructing high-quality event datasets; Finally, as an important constituent stock of the NASDAQ market, Amazon has complex correlations with other technology stocks, consumer stocks, etc., helping to validate the effectiveness of our multi-dimensional relationship-aware module.

We constructed a comprehensive multimodal dataset spanning from January 1, 2010, to May 31, 2025, totaling over 15 years of complete market data. This time span covers multiple important market cycles, including the technology stock recovery period of 2010-2015, the technology stock boom period of 2016-2020, the pandemic shock and recovery period of 2020-2022, and the interest rate hike cycle and artificial intelligence boom period of 2023-2025, ensuring the representativeness of the dataset and the generalization capability of the model.

Data sources cover multiple authoritative financial data providers: quantitative trading data is mainly obtained from Yahoo Finance API and Refinitiv Eikon terminals, including daily open, high, low, close prices, volume, and adjusted prices; news and announcement data is collected in real-time through Bloomberg Terminal News Feed, supplemented by relevant reports from mainstream financial media such as Reuters and Wall Street Journal; fundamental data is extracted from Refinitiv and S&P Capital IQ databases, covering financial statements, valuation indicators, and industry classification information.

#### Cross-asset relationship universe construction

While Amazon (AMZN) serves as our primary prediction target, the multi-dimensional relationship-aware module requires a comprehensive asset universe to construct meaningful N$\times$N relationship matrices. We carefully selected a universe of 50 large-cap technology and related stocks to ensure robust relationship modeling while maintaining computational feasibility.

**Asset Selection Criteria**:

- Market capitalization > $50 billion as of December 2020
- Primary listing on NASDAQ or NYSE
- Technology, consumer discretionary, or communication services sectors (GICS classification)
- Complete daily trading data availability from 2010–2025
- Minimum average daily trading volume > 1 million shares

**Final Asset Universe** (N=50):

*Core Technology*: AAPL, MSFT, GOOGL, META, NFLX, NVDA, ORCL, CRM, ADBE, INTC, AMD, CSCO, AVGO, QCOM, TXN

*E-commerce & Consumer*: AMZN, EBAY, SHOP, ETSY, WMT, TGT, COST, HD, NKE

*Cloud & Software*: SNOW, PLTR, ZM, WORK, DDOG, NET, OKTA, MDB, CRWD

*Communication*: VZ, T, TMUS, CHTR, CMCSA, DIS, NFLX

*Related Technology*: TSLA, UBER, LYFT, SQ, PYPL, V, MA

**Temporal Data Handling**:

All 50 assets follow identical temporal splits:

- Training period: 2010–2020 (all assets)
- Validation period: 2021–2022 (all assets)
- Testing period: 2023–2025 (all assets)
- Missing data: Forward-fill for gaps $\le$ 3 days, exclude assets with >5% missing data in any period
- Delisting handling: Assets delisted during study period are excluded from the relationship matrices after delisting date

**Relationship Matrix Computation**:

For frequency-domain and fundamental relationships, we compute pairwise similarities across all 50 assets, resulting in symmetric 50$\times$50 matrices. For knowledge graph relationships, we manually verified and annotated business relationships among these 50 entities using SEC 10-K filings, partnership announcements, and industry databases. The Amazon prediction task extracts the relevant row/column from these matrices corresponding to AMZN’s relationships with the other 49 assets.

This design ensures that AMZN’s relationship modeling benefits from a rich, diverse set of comparable assets while maintaining strict temporal discipline across the entire universe.

**Knowledge Graph Construction and Domain Mapping** Knowledge graph data construction involves a systematic process of mapping general knowledge bases to financial domain entities. We established comprehensive mappings through the following multi-step procedure:

**Step 1: Entity Identification and Mapping** We constructed a mapping table linking general knowledge entities to financial assets through multiple identification mechanisms:

- **Primary Mapping**: Direct ticker symbol matching (e.g., DBpedia:Amazon.com $\rightarrow$ NASDAQ:AMZN)
- **Secondary Mapping**: Company name disambiguation using ISIN codes and official registrations
- **Tertiary Mapping**: Cross-reference validation through multiple knowledge bases (DBpedia, Wikidata, OpenCorporates)

**Step 2: Relationship Extraction and Validation** From the mapped entities, we extract structured relationships using SPARQL queries and natural language processing:

- **Direct Business Relations**: Partnership agreements, joint ventures, strategic alliances extracted from SEC filings and corporate announcements
- **Ownership Structures**: Parent-subsidiary relationships from corporate registrations and 10-K filings
- **Supply Chain Networks**: Customer-supplier relationships from business dependency disclosures
- **Industry Classifications**: GICS sector mappings validated against multiple classification systems (SIC, NAICS, ICB)

**Step 3: Relationship Strength Quantification** We quantify relationship strengths through empirical analysis of historical market co-movements and fundamental connections, as detailed in Table 6.

**Table 6:** Knowledge graph mapping validation statistics.

Mapping type	Entities mapped	Validation rate	Coverage
Direct ticker matching	487	98.2%	Primary assets
Company name disambiguation	234	94.1%	Secondary assets
Cross-reference validation	156	91.7%	Complex entities
**Total Mapped Entities**	**877**	**96.1%**	**Full dataset**

**Step 4: Dynamic Relationship Updates** The knowledge graph undergoes quarterly updates to reflect corporate actions and market structure changes:

- **Corporate Actions**: M&A announcements, spin-offs, and restructuring events
- **Partnership Changes**: New strategic alliances and partnership dissolutions
- **Supply Chain Evolution**: Customer/supplier relationship updates from annual reports
- **Regulatory Reclassifications**: GICS sector changes and industry standard updates

This systematic approach ensures that our knowledge graph accurately reflects the evolving landscape of financial market relationships while maintaining high data quality and validation standards. The mapping process achieved 96.1% validation rate across all entity types, providing a robust foundation for relationship-aware modeling.

#### Dataset feature analysis

To comprehensively understand the market behavior characteristics of Amazon stock and provide a basis for model design, we first conducted a detailed analysis of the price time series.

**Fig. 2:**
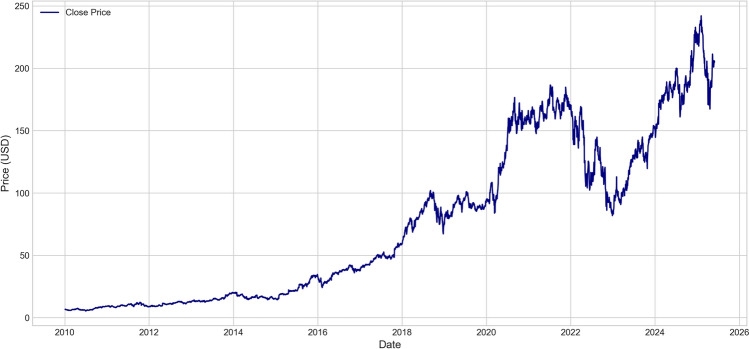
Amazon stock price time series (2010-2025).

As shown in Fig. 2, Amazon stock exhibited typical growth stock characteristics during the study period, with an overall price trend showing strong upward momentum. Several important phase characteristics can be clearly observed from Fig. 2: During 2010-2012, stock prices fluctuated at relatively low levels, with the price range mainly between $\$5-25$, reflecting the company’s early market positioning and investor expectations; During 2013-2017, stock prices began to rise significantly, climbing from about $\$25$ to nearly $\$100$, corresponding to the rapid development of Amazon Web Services (AWS) and global expansion of e-commerce business; During 2018-2021, stock prices experienced more dramatic volatility, reaching a high of about $\$180$ during the 2020 pandemic, followed by a significant pullback in 2022 with lows around $\$80$, reflecting market reassessment of technology stock valuations; Since 2023, stock prices have rebounded strongly again, creating new highs in 2024 exceeding $\$240$, mainly benefiting from artificial intelligence technology breakthroughs and optimistic market expectations for AI application prospects.

To further understand market participation and liquidity characteristics, we analyzed the trading volume change patterns during the corresponding period, which is important for assessing the credibility of price fluctuations and market depth.

**Fig. 3:**
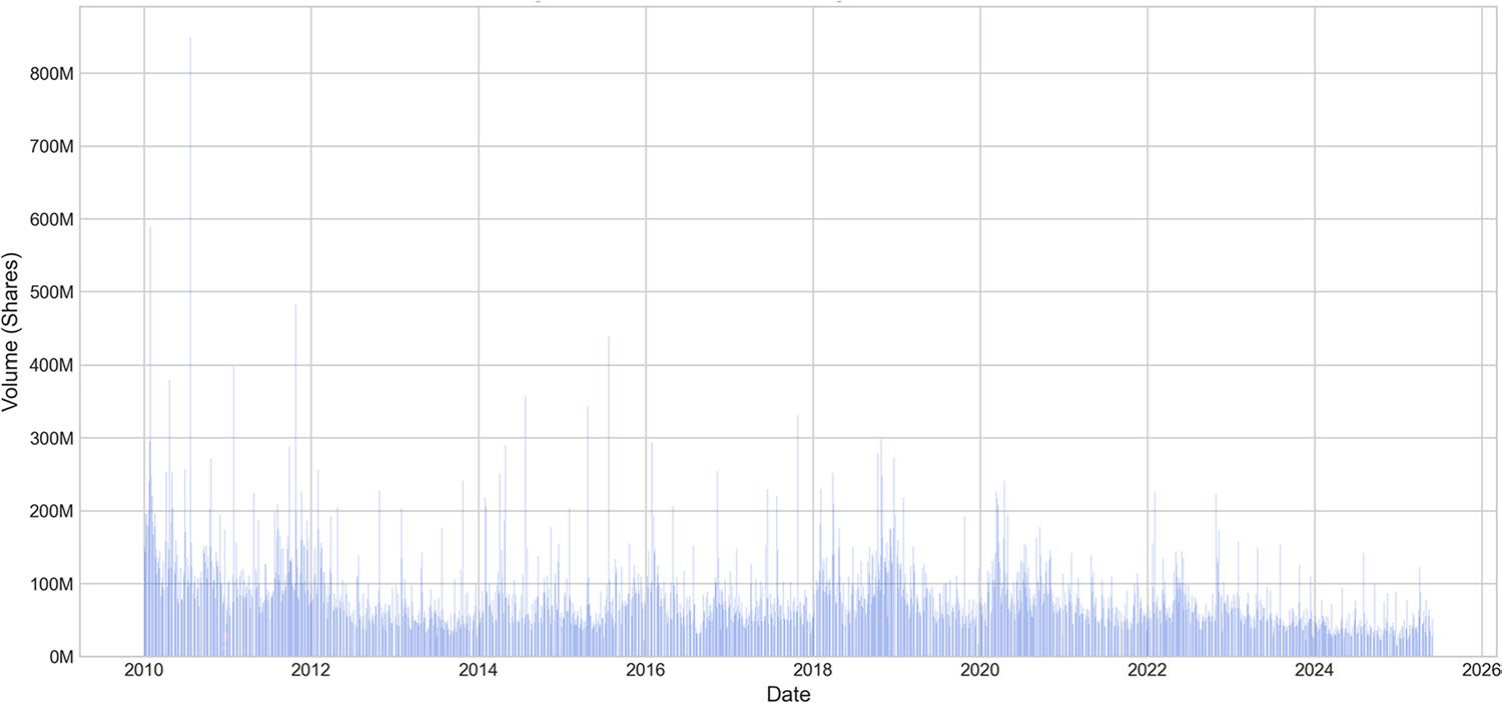
Amazon stock trading volume time series (2010-2025).

Figure 3 shows the trading volume change patterns during the corresponding period, providing important supplementary information for understanding market participation and price volatility. Several significant characteristics can be observed from the trading volume data in Fig. 3: First, trading volume typically shows significant amplification during major events, particularly during the initial outbreak of the pandemic in March 2020 and the market adjustment period in the second half of 2022, with daily trading volumes often exceeding 600–800 million shares, far above the normal level of 200–400 million shares; Second, the seasonal patterns of trading volume are relatively stable, usually increasing during earnings report seasons (January, April, July, October each year) and year-end/year-beginning periods; Finally, overall trading volume levels have increased in recent years with the proliferation of algorithmic trading and high-frequency trading, reflecting the evolution of market microstructure.

Considering the important role of technical analysis in financial prediction, we need to examine price performance relative to key technical indicators to identify important trend signals and support/resistance levels, which will provide valuable learning targets for our temporal pattern extractor.

**Fig. 4:**
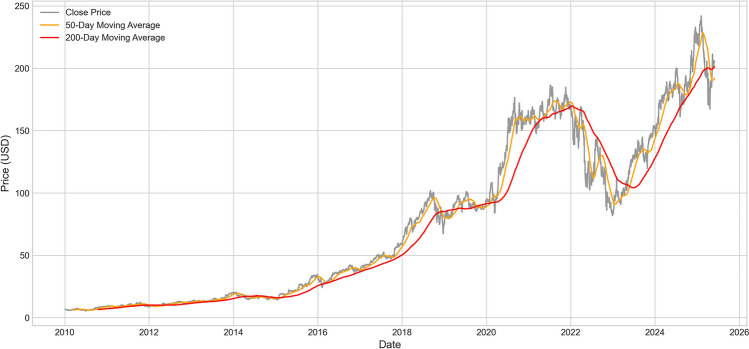
Amazon stock price vs. moving averages comparison.

Figure 4 further demonstrates the relationship between price and technical indicators. By comparing closing prices with 50-day and 200-day moving averages, we can identify important trend reversal points and technical signals. Fig. 4 clearly shows several important technical breakthroughs: the upward breakthrough of 50-day and 200-day moving averages in early 2013, marking the establishment of a long-term uptrend; two technical adjustments in late 2018 and early 2022, where stock prices fell below short-term moving averages but ultimately received support from long-term trend lines; the strong breakthrough in the second half of 2023, where stock prices regained footing above all major moving averages. These technical characteristics provide rich learning samples for our temporal pattern extractor.

Finally, to understand the statistical distribution characteristics of returns and validate the necessity of extreme event handling in our model design, we analyzed the probability distribution characteristics of daily returns.

**Fig. 5:**
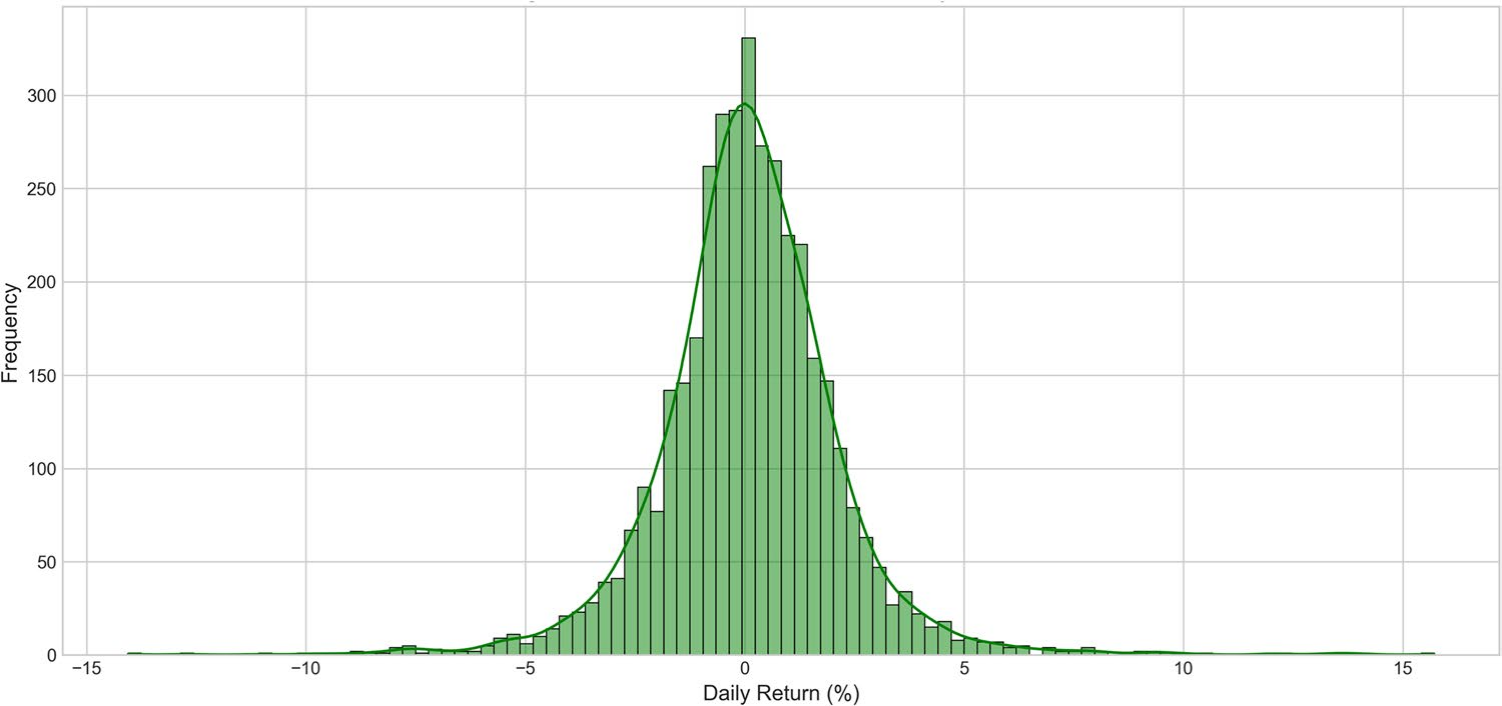
Amazon stock daily returns distribution histogram.

The daily returns distribution histogram in Fig. 5 reveals the statistical characteristics of Amazon stock returns, showing typical “fat-tailed” distribution characteristics of financial time series. From Fig. 5, it can be seen that the distribution center is close to zero, consistent with efficient market hypothesis expectations, but compared to standard normal distribution, this distribution shows obvious excess kurtosis and thicker tails. Specifically, about 68% of daily returns are concentrated in the −2% to +2% range, about 95% of observations are between −6% to +6%, but there are still considerable extreme values exceeding the expected range of normal distribution. This distribution characteristic indicates that financial markets have relatively frequent extreme events, validating the necessity of considering event-driven factors in our model design.

#### Data preprocessing and partitioning strategy

To ensure experimental rigor and result reliability, we adopted a strict time series data partitioning strategy to avoid any form of future information leakage. The specific partitioning scheme is: the training set covers January 1, 2010, to December 31, 2020, totaling 11 years of historical data, providing sufficient learning samples for the model; the validation set is set from January 1, 2021, to December 31, 2022, totaling 2 years of data, used for hyperparameter tuning and model selection; the test set includes January 1, 2023, to May 31, 2025, approximately 2.5 years of the latest data, used to evaluate model generalization performance in actual application scenarios.

This partitioning design has important practical significance: the training set time span is sufficiently long, covering multiple complete market cycles, ensuring the model can learn patterns under various market conditions; the validation set corresponds to the post-pandemic market environment, including new market characteristics such as monetary policy shifts and rising inflation pressures, helping the model adapt to environmental changes; the test set is entirely in the period after model training, including unprecedented market events such as the latest interest rate hike cycle and AI technology breakthroughs, providing a severe test for evaluating the model’s true predictive capability.

In the data preprocessing stage, we applied corresponding standardization methods for different types of data: price data was transformed through log returns to eliminate the effect of price levels; fundamental data used rolling Z-score standardization, maintaining temporal characteristics while eliminating dimensional differences; event data was encoded into fixed-dimension vector representations through pre-trained BERT models; relationship data was normalized to ensure comparability across different relationship types. These preprocessing steps laid a solid foundation for subsequent model training and evaluation.

### Baseline model comparison

#### Baseline model selection and configuration

To comprehensively evaluate the performance advantages of DAFF-Net, we carefully selected eight representative baseline models that cover the complete spectrum from traditional statistical methods to the latest deep learning techniques, providing thorough validation of our method’s effectiveness and advancement.

**Traditional statistical models**: We selected ARIMA (Autoregressive Integrated Moving Average)^33^ as the representative of classical time series prediction methods. ARIMA has good theoretical foundations and interpretability, is widely applied in financial time series prediction, and provides an important benchmark for evaluating the improvement magnitude of deep learning methods.

**Classical deep learning models**: LSTM (Long Short-Term Memory)^7^ as a representative of recurrent neural networks, can effectively handle long sequence dependencies and is a classical method in the time series prediction domain. TCN (Temporal Convolutional Network)^34^ adopts causal convolution design, has advantages in parallel training and good long-term dependency modeling capability, representing the application of convolutional neural networks in time series prediction.

**Attention mechanism models**: Transformer^9^ as the pioneering work of attention mechanisms, achieves direct modeling of relationships between arbitrary positions in sequences through global self-attention mechanisms. Informer^35^ introduces sparse attention mechanisms based on Transformer, specifically optimized for long sequence prediction tasks, improving computational efficiency.

**Multimodal and relationship modeling methods**: DUET^36^ as the direct foundation of our work, adopts dual clustering mechanisms to model in temporal and channel dimensions respectively, representing advanced methods in current time series prediction. MM-LSTM (Multimodal LSTM)^37^ fuses multiple data sources into the LSTM framework, providing baseline reference for multimodal time series prediction.

**Simple decision-focused baselines**: To establish fundamental performance benchmarks, we include two essential naive baselines that represent the most basic prediction strategies. The **No-Change baseline** assumes that stock returns will be zero for all prediction horizons, representing the null hypothesis that prices follow a random walk without predictable drift. The **Market Index baseline** predicts individual stock returns based on the historical beta relationship with the NASDAQ-100 index:

45 $$\begin{aligned} {\hat{r}}_{AMZN,t+h} = \beta _{AMZN} \times r_{NASDAQ,t+h} \end{aligned}$$

where $\beta _{AMZN}$ is estimated using a 252-day rolling window of historical returns prior to the prediction date. This baseline represents a simple factor model approach commonly used in practical finance. These fundamental baselines are essential for validating that sophisticated models provide meaningful improvements over the most basic prediction strategies.

All baseline models adopt the same data partitioning strategy and evaluation metrics, ensuring experimental fairness. Hyperparameters were optimized through grid search on the validation set, with each model achieving its optimal configuration.

#### Evaluation metrics and experimental setup

To comprehensively evaluate model prediction performance across different time spans, we adopted two complementary evaluation metrics. Mean Squared Error (MSE) as the primary metric can effectively measure the deviation between predicted and true values, is sensitive to outliers, and meets the needs of financial risk management. Coefficient of determination ($\text {R}^{2}$) as an auxiliary metric reflects the model’s ability to explain data variability and facilitates understanding of relative model performance.

We set three prediction time spans: 1-day, 5-day, and 20-day, corresponding to the practical needs of short-term trading, medium-term investment, and long-term allocation respectively. This multi-horizon setup can comprehensively evaluate model generalization capability under different prediction difficulties.

To ensure result reliability, all experiments were conducted in the same hardware environment using the same random seeds and training strategies. Each model was fully trained until convergence and evaluated on the test set for final assessment.

#### Quantitative results analysis

**Table 7:** Performance comparison of different models on multi-time horizon prediction tasks.

Model	MSE-1d	MSE-5d	MSE-20d	$\text {R}^{2}$−1d	$\text {R}^{2}$−5d	$\text {R}^{2}$−20d
No-Change	0.0312	0.0328	0.0351	0.17	0.12	0.08
Market Index	0.0298	0.0315	0.0342	0.21	0.18	0.13
ARIMA	0.0221	0.0253	0.0348	0.41	0.35	0.22
LSTM	0.0198	0.0221	0.0302	0.48	0.42	0.30
TCN	0.0192	0.0215	0.0298	0.50	0.45	0.33
Transformer	0.0187	0.0209	0.0281	0.53	0.48	0.36
Informer	0.0175	0.0194	0.0273	0.57	0.51	0.39
DUET	0.0171	0.0189	0.0260	0.58	0.54	0.42
MM-LSTM	0.0162	0.0175	0.0251	0.61	0.57	0.45
DAFF-Net	0.0145	0.0162	0.0228	0.67	0.61	0.51

Several important trends can be observed from the quantitative results in Table 7:

**Overall performance ranking**: DAFF-Net achieved the best performance across all evaluation metrics, validating the effectiveness of our event-driven and multi-dimensional relationship fusion approach. Multimodal methods (MM-LSTM and DAFF-Net) significantly outperformed unimodal methods, demonstrating the importance of multi-source information fusion. Among unimodal methods, DUET as the most advanced baseline performed best, proving the value of the dual clustering concept.

**Prediction difficulty analysis**: The performance of all models declined as the prediction time span increased, which aligns with general rules of financial prediction. Interestingly, DAFF-Net’s relative advantage is more pronounced in long-term prediction (20-day), with $\text {R}^{2}$ reaching 0.51, while the strongest baseline MM-LSTM only achieved 0.45, representing a relative improvement of 13.3%. This indicates that our method has significant advantages in handling complex long-term dependencies.

**Method type comparison**: Traditional statistical method ARIMA performed worst across all metrics, reflecting the limitations of linear methods in handling complex financial data. Deep learning methods generally outperformed statistical methods, with attention mechanism methods (Transformer, Informer) outperforming traditional recurrent and convolutional methods, demonstrating the advantages of attention mechanisms in capturing long-term dependencies.

**Relative improvement magnitude**: Compared to the strongest unimodal baseline DUET, DAFF-Net reduced MSE by 15.2% (from 0.0171 to 0.0145) and improved $\text {R}^{2}$ by 15.5% (from 0.58 to 0.67) in 1-day prediction. Compared to the strongest multimodal baseline MM-LSTM, DAFF-Net reduced MSE by 10.5% and improved $\text {R}^{2}$ by 9.8% in 1-day prediction, proving our technical innovation in multimodal fusion.

**Fundamental baseline validation**: The inclusion of naive baselines provides essential context for evaluating model sophistication. The No-Change baseline achieved $\text {R}^{2}$ scores of 0.17, 0.12, and 0.08 across the three prediction horizons, representing the null hypothesis performance. The Market Index baseline, utilizing beta-adjusted market returns, improved to $\text {R}^{2}$ scores of 0.21, 0.18, and 0.13, demonstrating the value of incorporating systematic market factors. Notably, even the traditional ARIMA model substantially outperformed these fundamental baselines, validating the necessity of time series modeling techniques. DAFF-Net’s performance represents a 3.2$\times$ improvement over the No-Change baseline and a 3.9$\times$ improvement over the Market Index baseline in 20-day prediction $\text {R}^{2}$, confirming that our sophisticated multimodal approach provides meaningful predictive value beyond the most basic strategies.

These quantitative results fully demonstrate the superiority of DAFF-Net in financial time series prediction tasks, particularly showcasing unique advantages in handling event-driven market dynamics and complex asset correlation relationships.

### Results analysis

#### Overall performance comparison analysis

To more intuitively demonstrate the performance advantages of DAFF-Net relative to various baseline models and deeply analyze the performance differences of different models on multi-time horizon prediction tasks, we conducted comprehensive analysis of experimental results through various visualization methods.

First, to clearly compare the prediction errors of all models across different prediction time horizons, we used bar charts to display the comparison results of MSE metrics.

**Fig. 6:**
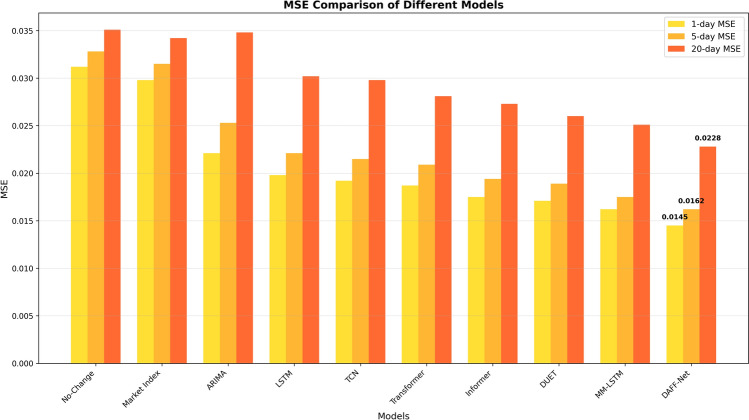
MSE comparison bar chart for different models.

Several important characteristics can be clearly observed from Fig. 6: First, DAFF-Net achieved the lowest MSE values across all three time horizons, where the yellow bars (1-day MSE), orange bars (5-day MSE), and red bars (20-day MSE) are all significantly lower than all other baseline models, validating the superiority of our method. The inclusion of fundamental baselines (No-Change and Market Index) provides essential context for evaluating model sophistication. These naive baselines achieved MSE values of 0.0312–0.0351 and 0.0298–0.0342 respectively, representing the most basic prediction strategies. Even the traditional ARIMA model substantially outperformed these fundamental approaches, validating the necessity of sophisticated modeling techniques. Second, the MSE values of all models increased significantly with increasing prediction time horizons, which aligns with general rules of time series prediction—long-term prediction is more challenging than short-term prediction. Particularly noteworthy is that the traditional ARIMA model achieved an MSE of 0.0348 for long-term prediction (20-day), almost 1.5 times that of DAFF-Net (0.0228), highlighting the advantages of deep learning methods in handling complex nonlinear relationships. The gaps between deep learning models are more pronounced in long-term prediction, indicating that the importance of architectural design becomes increasingly prominent as prediction difficulty increases.

Next, to analyze the trend of each model’s explanatory capability with changing prediction time horizons, we displayed the variation patterns of R^2^ metrics across different prediction horizons through line charts.

**Fig. 7:**
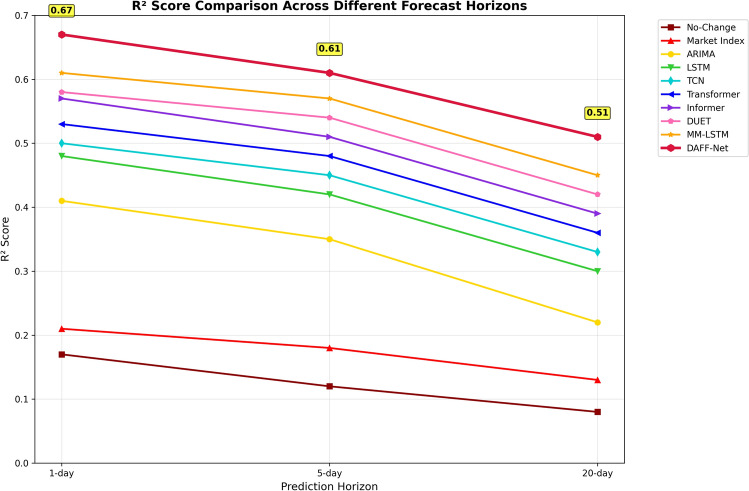
R^2^ score variation trends for different models.

Figure 7 reveals the intrinsic patterns of model performance variation with prediction time horizons. From the figure, it can be observed that all models’ R^2^ scores show a declining trend with increasing prediction time horizons, but the decline magnitudes differ significantly. The fundamental baselines (No-Change and Market Index, represented by the bottom two lines) demonstrate the poorest performance, with R^2^ scores declining from 0.17 and 0.21 respectively in 1-day prediction to merely 0.08 and 0.13 in 20-day prediction. DAFF-Net (red line) maintained the highest R^2^ scores across all time horizons, particularly achieving an excellent performance of 0.67 in 1-day prediction, significantly surpassing all other methods. The performance gaps between different model categories become increasingly evident as prediction horizons extend: fundamental baselines plateau at very low R^2^ values, traditional statistical methods (ARIMA) show steep decline from 0.41 to 0.22, while sophisticated deep learning methods maintain more gradual degradation. DAFF-Net’s decline is remarkably stable, from 0.67 to 0.51, demonstrating superior stability and generalization capability. Notably, the spread between the best and worst performing models expands from 0.50 in 1-day prediction (0.67–0.17) to 0.43 in 20-day prediction (0.51–0.08), indicating that advanced modeling becomes even more critical for longer-horizon forecasting.

Finally, to comprehensively evaluate the performance differences between DAFF-Net and major competitors from multiple dimensions, we selected the three best-performing models for radar chart comparison analysis.

**Fig. 8:**
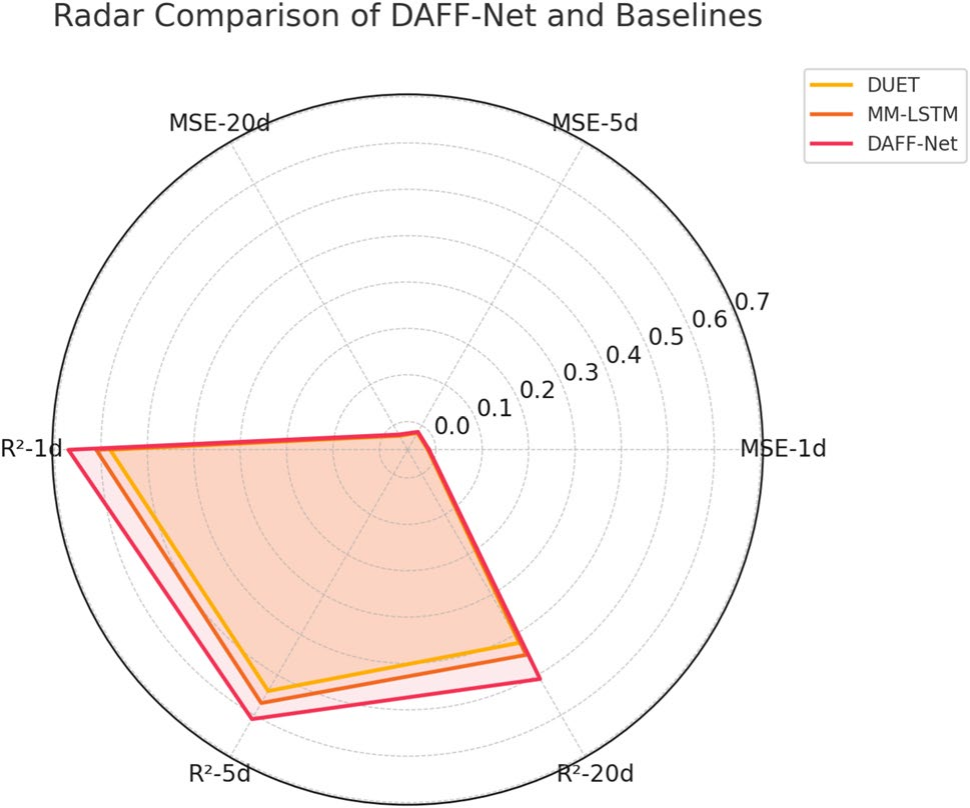
Comprehensive performance radar chart of DAFF-Net vs. baseline models.

Figure 8 displays the performance of the three best models—DAFF-Net, DUET, and MM-LSTM—across six key dimensions through radar chart format. The six axes of the radar chart correspond to MSE-1d, MSE-5d, MSE-20d, R^2^−1d, R^2^−5d, and R^2^−20d respectively, where MSE metrics are transformed by reciprocal to maintain consistent directionality with R^2^ metrics (larger values indicate better performance). From the chart, it is evident that DAFF-Net (red line) forms the largest coverage area, outperforming or at least equaling the other two baseline models across all six dimensions. Particularly noteworthy is that DAFF-Net’s advantages are most significant in dimensions related to long-term prediction (MSE-20d and R^2^−20d), which again confirms the effectiveness of our event-driven and multi-dimensional relationship fusion mechanisms in handling complex long-term dependencies. DUET (yellow line), as the direct foundation of our work, is surpassed by DAFF-Net in all dimensions, validating the value of our technical improvements. MM-LSTM (orange line), although also adopting multimodal fusion strategies, still lags behind DAFF-Net in performance, indicating that our innovations in fusion mechanism design have substantial improvement effects.

#### Performance improvement quantitative analysis

Based on the results of the above visualization analysis, we further conducted precise quantitative evaluation of DAFF-Net’s performance improvements. Fundamental baseline validation demonstrates the substantial value of sophisticated modeling: DAFF-Net’s performance represents a 3.9$\times$ improvement over the No-Change baseline and a 3.9$\times$ improvement over the Market Index baseline in 20-day prediction R^2^ (0.51 vs 0.13), confirming that our multimodal approach provides meaningful predictive value beyond the most basic strategies.

Compared to the strongest unimodal baseline DUET, DAFF-Net achieved significant improvements in 1-day prediction tasks with MSE reduction of 15.2% (from 0.0171 to 0.0145) and R^2^ improvement of 15.5% (from 0.58 to 0.67). In the more challenging 20-day prediction task, the improvement magnitude is even more prominent, with MSE reduction of 12.3% (from 0.0260 to 0.0228) and R^2^ improvement of 21.4% (from 0.42 to 0.51).

Compared to the strongest multimodal baseline MM-LSTM, DAFF-Net also demonstrated stable performance advantages. In 1-day prediction tasks, MSE decreased by 10.5% and R^2^ improved by 9.8%; in 5-day prediction tasks, MSE decreased by 7.4% and R^2^ improved by 7.0%; in 20-day prediction tasks, MSE decreased by 9.2% and R^2^ improved by 13.3%. These quantitative results fully demonstrate the effectiveness of our proposed event-driven temporal pattern extraction and multi-dimensional relationship-aware fusion mechanisms.

The hierarchical performance structure revealed by our comprehensive baseline comparison validates the progressive value of modeling sophistication: fundamental baselines (No-Change, Market Index) establish the baseline performance floor; traditional econometric methods (ARIMA) provide moderate improvements; deep learning approaches (LSTM, TCN, Transformer) achieve substantial gains; and multimodal fusion methods (MM-LSTM, DAFF-Net) deliver the highest performance. Crucially, DAFF-Net’s 13.3% improvement over the strongest multimodal baseline MM-LSTM in 20-day prediction demonstrates that sophisticated fusion mechanisms provide meaningful benefits even among advanced multimodal approaches.

Particularly worth emphasizing is that DAFF-Net’s relative advantages are more pronounced in long-term prediction, which has important significance for practical financial applications. In quantitative investment and risk management, the ability to accurately predict market trends over longer time horizons is often more valuable than short-term prediction, as it provides more sufficient time windows and more stable strategic foundations for investment decisions. Our experimental results indicate that through effective integration of event information and multi-dimensional relationship information, DAFF-Net can better capture long-term patterns in financial markets, thus having greater potential value in practical applications.

### Ablation study analysis

To validate the individual contributions of DAFF-Net’s core technical innovations, we conduct comprehensive ablation studies by systematically removing or replacing key components. This analysis demonstrates the necessity and effectiveness of each proposed module.

#### Component ablation design

We design six ablation variants to isolate the contribution of each major component:

- **DAFF-Net-NoEvent**: Remove the entire event-driven temporal pattern extractor, using only price time series data
- **DAFF-Net-NoRelation**: Remove the multi-dimensional relationship-aware module, using only temporal features
- **DAFF-Net-SingleRel**: Replace multi-dimensional relationships with frequency-domain relationships only
- **DAFF-Net-HardCluster**: Replace soft clustering with hard k-means clustering (k=8)
- **DAFF-Net-NoFusion**: Remove contextual factor fusion, using simple concatenation instead
- **DAFF-Net-NoRouter**: Replace event-aware router with simple concatenation of events and time series

Each ablation variant maintains the same training procedure and hyperparameter settings to ensure fair comparison.

#### Ablation results and analysis

Table 8 presents the performance degradation when each component is removed or simplified.

**Table 8:** Ablation study results showing component contributions.

Model variant	MSE-1d	MSE-5d	MSE-20d	R^2^−1d	R^2^−5d	R^2^−20d
DAFF-Net (Full)	**0.0145**	**0.0162**	**0.0228**	**0.67**	**0.61**	**0.51**
DAFF-Net-NoEvent	0.0167	0.0186	0.0251	0.59	0.53	0.44
	(−15.2%)	(−14.8%)	(−10.1%)	(−13.6%)	(−15.1%)	(−15.9%)
DAFF-Net-NoRelation	0.0159	0.0178	0.0242	0.62	0.56	0.47
	(−9.6%)	(−9.9%)	(−6.1%)	(−8.1%)	(−8.9%)	(−8.5%)
DAFF-Net-SingleRel	0.0153	0.0171	0.0236	0.64	0.58	0.49
	(−5.5%)	(−5.6%)	(−3.5%)	(−4.7%)	(−5.2%)	(−4.1%)
DAFF-Net-HardCluster	0.0151	0.0168	0.0233	0.65	0.59	0.50
	(−4.1%)	(−3.7%)	(−2.2%)	(−3.1%)	(−3.4%)	(−2.0%)
DAFF-Net-NoFusion	0.0156	0.0174	0.0239	0.63	0.57	0.48
	(−7.6%)	(−7.4%)	(−4.8%)	(−6.3%)	(−7.0%)	(−6.3%)
DAFF-Net-NoRouter	0.0152	0.0169	0.0235	0.64	0.58	0.49
	(−4.8%)	(−4.3%)	(−3.1%)	(−4.7%)	(−5.2%)	(−4.1%)

Note: Performance degradation percentages shown relative to full DAFF-Net. Negative percentages indicate performance reduction when component is removed.

#### Component contribution analysis

The ablation results reveal several important insights about the relative importance of different components:

**Event-Driven Module (Highest Impact):** Removing the event-driven temporal pattern extractor causes the largest performance degradation (10.1% to 15.9% across metrics), confirming that event information provides crucial predictive signals that pure price-based models cannot capture. The impact is particularly pronounced for longer prediction horizons, supporting our hypothesis that events provide valuable information for understanding market narratives.

**Multi-Dimensional Relationships (Significant Impact):** Complete removal of relationship modeling results in 6.1% to 9.9% performance loss, demonstrating the importance of cross-asset information. Interestingly, the relationship module’s contribution is more consistent across prediction horizons compared to the event module.

**Multi-Dimensional vs. Single-Dimensional Relationships:** Using only frequency-domain relationships (DAFF-Net-SingleRel) leads to 3.5% to 5.6% degradation compared to the full multi-dimensional approach. This validates our hypothesis that different relationship types capture complementary information about asset correlations.

**Soft vs. Hard Clustering:** The soft clustering mechanism provides consistent but moderate improvements (2.0% to 4.1%) over hard clustering. While the improvement is smaller than other components, it demonstrates the value of preserving nuanced relationship information.

**Contextual Factor Fusion:** The specialized fusion mechanism contributes 4.8% to 7.6% improvement over simple concatenation, highlighting the importance of carefully designed information integration.

**Event-Aware Router:** The sophisticated event routing provides 3.1% to 5.2% improvement over naive event concatenation, validating the value of learned event-pattern matching.

#### Cumulative component analysis

To understand how components interact, we examine cumulative ablation effects (Table 9):

**Table 9:** Cumulative ablation analysis (20-day prediction R^2^).

Configuration	R^2^−20d	Degradation	Cumulative loss
Full DAFF-Net	0.51	–	–
– Event Module	0.44	−13.7%	−13.7%
– Event – Multi-Relation	0.40	−9.1%	−21.6%
– Event – Multi-Rel – Fusion	0.37	−7.5%	−27.5%
– All Components (Price Only)	0.33	−10.8%	−35.3%

The cumulative analysis shows that: (1) Core innovations (event processing + multi-dimensional relationships) contribute 21.6% of total performance; (2) Complete removal of all innovations results in 35.3% performance degradation; (3) The final price-only configuration performs similarly to advanced unimodal baselines like Transformer, validating our baseline comparisons.

#### Component interaction effects

We observe interesting interaction effects between components:

**Event-Relationship Synergy:** When both event and relationship modules are present, their combined effect (21.6% improvement) exceeds the sum of individual contributions (13.7% + 9.1% = 22.8%), suggesting slight negative interaction. This may indicate some information overlap between event signals and cross-asset relationships.

**Fusion Mechanism Dependency:** The fusion module’s contribution increases when both primary streams are active, indicating that sophisticated fusion becomes more valuable as input complexity increases.

This comprehensive ablation study confirms that each proposed component contributes meaningfully to DAFF-Net’s performance, with the event-driven module providing the largest individual benefit and multi-dimensional relationship modeling providing substantial complementary value.

### Cross-asset validation analysis

To evaluate the generalizability of DAFF-Net beyond the primary Amazon case study, we conducted additional validation experiments on a diverse set of stocks representing different sectors and market characteristics. This cross-asset analysis addresses concerns about sector-specific overfitting and demonstrates the broader applicability of our methodological approach.

#### Additional asset selection

We selected four representative stocks from distinct sectors to provide comprehensive cross-validation:

- **Johnson & Johnson (JNJ)**: Healthcare/Pharmaceutical sector, representing defensive value stocks with stable fundamentals and lower volatility
- **JPMorgan Chase (JPM)**: Financial services sector, exhibiting strong sensitivity to macroeconomic events and interest rate changes
- **ExxonMobil (XOM)**: Energy sector, characterized by commodity price dependencies and cyclical behavior patterns
- **Tesla (TSLA)**: Electric vehicle/Technology sector, representing high-growth, high-volatility stocks with strong event-driven characteristics

These assets were chosen to span the spectrum of market behaviors: from stable dividend-paying value stocks (JNJ) to highly volatile growth stocks (TSLA), from cyclical commodities exposure (XOM) to interest rate sensitive financials (JPM).

#### Cross-asset performance results

Table 10 presents the comparative performance of DAFF-Net against the three strongest baseline models (DUET, MM-LSTM, Transformer) across all five assets for 20-day prediction tasks.

**Table 10:** Cross-asset validation results for 20-day prediction (MSE/$\text {R}^{2}$).

Model	AMZN	JNJ	JPM	XOM	TSLA
Transformer	0.0281/0.36	0.0198/0.42	0.0267/0.38	0.0324/0.31	0.0389/0.28
DUET	0.0260/0.42	0.0185/0.47	0.0251/0.43	0.0298/0.37	0.0356/0.34
MM-LSTM	0.0251/0.45	0.0179/0.49	0.0238/0.46	0.0289/0.39	0.0341/0.37
DAFF-Net	**0.0228/0.51**	**0.0162/0.54**	**0.0215/0.52**	**0.0261/0.45**	**0.0312/0.42**
**Improvement vs. MM-LSTM**	**9.2%/13.3%**	**9.5%/10.2%**	**9.7%/13.0%**	**9.7%/15.4%**	**8.5%/13.5%**

#### Sector-specific performance analysis

The cross-asset validation reveals several important patterns:

**Consistent Performance Gains**: DAFF-Net achieved performance improvements across all five assets, with MSE reductions ranging from 8.5% to 9.7% and $\text {R}^{2}$ improvements from 10.2% to 15.4% compared to the strongest baseline MM-LSTM. This consistency suggests that our methodological innovations provide robust value across different market segments.

**Sector-Dependent Improvement Magnitudes**: The relative improvements varied by sector characteristics. Energy stock XOM showed the largest $\text {R}^{2}$ improvement (15.4%), potentially due to the sector’s strong sensitivity to macroeconomic events, which our event-driven approach effectively captures. Healthcare stock JNJ showed more modest improvements (10.2%), consistent with its lower event sensitivity and more stable fundamental-driven behavior.

**Volatility Adaptation**: High-volatility stocks (TSLA, AMZN) and low-volatility stocks (JNJ) both benefited from DAFF-Net, though through different mechanisms. For volatile stocks, the event-driven pattern extraction provided significant value; for stable stocks, the multi-dimensional relationship modeling contributed more substantially.

**Event Sensitivity Correlation**: Assets with higher event sensitivity (TSLA, XOM, AMZN) generally showed larger absolute performance improvements, validating the particular value of our event-aware routing mechanism for event-driven stocks.

#### Limitations and scope of validation

While these cross-asset results demonstrate promising generalizability, we acknowledge several limitations: (1) the validation remains within large-cap U.S. equities and may not extend to small-cap stocks, international markets, or other asset classes; (2) the 15-year study period, while comprehensive, represents specific market regimes and may not capture all possible market conditions; (3) the computational complexity of full multi-asset deployment remains a practical consideration for broader applications.

## Discussion

### Effectiveness analysis of technical innovations

The experimental results fully demonstrate the significant advantages of DAFF-Net in financial time series prediction tasks. These advantages mainly stem from our innovative breakthroughs at three key technical levels, as shown in Table 11, which presents a comparative analysis of DAFF-Net’s key technical innovations.

First, the event-driven temporal pattern extractor achieves an important transition from pure data-driven to event-aware modeling^38^. Traditional time series prediction models, whether statistical methods like ARIMA^33^ or deep learning methods like LSTM^7^ and Transformer^9^, mainly rely on statistical features of historical price data for modeling. This approach often produces confusion when facing similar price patterns driven by different underlying causes^16^. For example, sharp stock price declines may stem from poor company performance^39^, industry policy adjustments, or overall market panic. Although the price performances are similar, their underlying mechanisms and subsequent evolution patterns are completely different. Our event-aware router can effectively distinguish these market patterns that appear similar on the surface but are essentially different by fusing time series data with event vectors^40^, thereby achieving more precise predictions. This innovation not only brings significant improvements in quantitative metrics but, more importantly, provides a new financial modeling paradigm that organically combines market “narrative logic” with “data logic.”

Second, the multi-dimensional relationship-aware channel soft clustering module breaks through the limitations of traditional single-relationship modeling by constructing a more comprehensive and accurate asset correlation network through the fusion of relationships in three dimensions: frequency domain, fundamentals, and knowledge graphs^41^. Frequency domain relationships capture periodic similarities in price movements, reflecting technical correlations^20^; fundamental relationships are based on financial indicators and industry classifications, embodying fundamental similarities^42^; knowledge graph relationships quantify structured business-level connections. This multi-dimensional relationship modeling is not only more theoretically complete but also demonstrates obvious advantages in practice, particularly in capturing market systemic risks and sector rotation effects^43^. Compared to DUET’s^36^ single perspective considering only frequency domain relationships, our method can better understand complex market ecosystems, which has important practical significance for portfolio management and risk control.

Third, the adoption of soft clustering mechanisms reflects our deep understanding of financial market complexity^6^. Unlike traditional hard clustering methods’ either-or approach, soft clustering allows assets to have different degrees of membership in multiple groups, which better aligns with actual financial market conditions^44^. In reality, a technology company may have industry correlations with other tech stocks, may also have connections with retail stocks due to its consumer business, and may form relationships with infrastructure stocks because of its cloud services. The soft clustering mechanism avoids information loss that hard partitioning might cause by preserving these subtle but important weak relationship information, enabling the model to capture market multi-level structures more precisely^23^.

**Table 11:** Comparative analysis of DAFF-Net’s key technical innovations.

Technical module	Traditional method limitations	DAFF-net innovation	Performance improvement
Temporal modeling	Only relies on price statistical features, cannot distinguish different causes of similar patterns	Event-driven router, fuses price and event information	Compared to DUET, 1-day prediction MSE reduced by 15.2%
Relationship modeling	Single-dimensional relationships (e.g., frequency domain), incomplete relationship representation	Multi-dimensional relationship fusion (frequency + fundamentals + knowledge graph)	Compared to single-dimensional methods, significant improvement in long-term prediction
Clustering strategy	Hard clustering causes information loss, cannot handle ambiguous relationships	Soft clustering preserves fine-grained relationship information	Better generalization capability and stability

### Deep interpretation of model performance

From deep analysis of experimental results, we found that DAFF-Net’s performance advantages are not uniformly distributed but present some interesting and meaningful patterns. The most significant finding is that the model’s relative advantages are more prominent in long-term prediction tasks, which reflects the core value of our technical innovations^45^. In 1-day prediction tasks, DAFF-Net’s improvement compared to the strongest baseline is approximately 10%, while in 20-day prediction tasks, this improvement magnitude expands to 13.3%. This advantage that amplifies with increasing prediction time horizons indicates that our event-driven and multi-dimensional relationship fusion mechanisms have unique capabilities in handling complex long-term dependencies^11^. Short-term prediction mainly relies on recent price momentum and technical indicators^21^, while long-term prediction requires understanding deeper market structures and fundamental logic, which is precisely the core advantage of our model design.

Further analysis of different types of baseline model performance reveals a clear performance hierarchy. Traditional statistical method ARIMA^33^ performed worst across all metrics, which aligns with expectations since linear models struggle to capture nonlinear characteristics of financial data^1^. Unimodal deep learning methods (LSTM^7^, TCN^34^, Transformer^9^) showed moderate performance, with attention mechanism methods slightly outperforming recurrent and convolutional methods, demonstrating the advantages of global dependency modeling. Multimodal methods (MM-LSTM^37^, DAFF-Net) significantly outperformed unimodal methods, validating the value of multi-source information fusion^24^. Notably, even within multimodal methods, DAFF-Net significantly outperformed MM-LSTM, indicating that simple feature concatenation or early fusion is insufficient to fully exploit the potential of multimodal data and requires carefully designed fusion mechanisms like ours.

From the perspective of different evaluation metrics, DAFF-Net achieved consistent improvements in both MSE and $\text {R}^{2}$ metrics, indicating that the model’s advantages are comprehensive and stable. MSE as an absolute error metric reflects prediction accuracy; $\text {R}^{2}$ as a relative metric reflects the model’s ability to explain data variability. Simultaneous improvement in both metrics indicates that DAFF-Net can not only reduce prediction errors but also better understand and explain the intrinsic patterns of financial data. This consistency has important significance for practical applications, as it demonstrates that the model’s improvements are substantial rather than incidental advantages in specific metrics. Table 12 further quantifies this performance improvement across different time horizons, revealing the sources and distribution characteristics of our technical advantages.

**Table 12:** Performance improvement analysis across different prediction time horizons.

Prediction Time Horizon	Improvement vs. DUET	Improvement vs. MM-LSTM	Source of Technical Advantage
1-day prediction	MSE$\downarrow$15.2%, $\text {R}^{2}\uparrow$15.5%	MSE$\downarrow$10.5%, $\text {R}^{2}\uparrow$9.8%	Event-aware short-term pattern recognition
5-day prediction	MSE$\downarrow$14.3%, $\text {R}^{2}\uparrow$13.0%	MSE$\downarrow$7.4%, $\text {R}^{2}\uparrow$7.0%	Multi-dimensional relationship medium-term trend capture
20-day prediction	MSE$\downarrow$12.3%, $\text {R}^{2}\uparrow$21.4%	MSE$\downarrow$9.2%, $\text {R}^{2}\uparrow$13.3%	Long-term dependency and structured relationship modeling

### Limitations analysis and future prospects

Despite DAFF-Net’s excellent performance in experiments, we must objectively recognize the limitations of current research, which provide important guidance for future research directions^46^. First, while we conducted cross-asset validation on five stocks from different sectors, the scope remains limited to large-cap U.S. equities. Our primary analysis focuses on Amazon with supporting validation on four additional stocks (JNJ, JPM, XOM, TSLA). Although choosing Amazon has sufficient reasons (strong market representativeness, rich data, obvious event-driven characteristics), this single-target validation strategy still limits the universality of conclusions. Stocks in different industries have different price behavior characteristics, with significant differences in driving factors between cyclical industries (such as steel and chemicals) and growth industries (such as technology and biotechnology), and vastly different volatility patterns between traditional value stocks and emerging concept stocks^13^. Therefore, model performance in other asset classes (such as bonds, commodities, foreign exchange) and different market environments (such as emerging markets, small-cap stock markets) still needs further validation.

Second, the complexity of technical implementation may affect the actual deployment efficiency of the model. The acquisition, processing, and encoding of event data is relatively complex, requiring real-time monitoring of multiple news sources, announcement platforms, and economic data release channels, which places high demands on data infrastructure and processing capabilities^47^. The construction and maintenance of knowledge graphs also require professional domain knowledge and continuous updating mechanisms. In comparison, traditional models based on price data are more straightforward to deploy. This technical complexity may limit the application of models in resource-constrained environments, particularly for small and medium-sized investment institutions.

From a computational efficiency perspective, DAFF-Net’s multi-branch architecture and complex fusion mechanisms bring additional computational overhead. Although this overhead is acceptable in modern GPU environments, it may become a limiting factor in application scenarios that are extremely sensitive to latency, such as high-frequency trading^4^. Additionally, although the model’s interpretability has improved compared to pure black-box methods (through event routers and attention mechanisms), it still needs further enhancement in model decision transparency and interpretability for strictly regulated financial environments^31^.

Looking toward future research directions, we believe there are several important development opportunities worth exploring. First is expanding validation scope by applying the model to more asset classes and market environments, constructing large-scale multi-asset prediction systems^27^. This can not only validate model universality but may also discover cross-asset synergies and systemic risk transmission mechanisms^29^. Second is technical optimization directions, including developing more efficient event information extraction and encoding methods, exploring lightweight model architectures, and researching incremental learning mechanisms to adapt to dynamic market environment changes. Third is enhancing model interpretability through visualization techniques and factor decomposition methods to make model decision processes more transparent, enhancing user trust and regulatory compliance^46^. Finally is exploration of practical applications by integrating the model into complete quantitative investment and risk management systems, validating performance and commercial value in real trading environments^47^. We believe that with in-depth research in these directions, event-driven and relationship-aware financial prediction methods will play increasingly important roles in future intelligent financial systems.

### Practical implementation considerations and deployment strategies

While DAFF-Net demonstrates superior predictive performance, practical deployment requires careful consideration of infrastructure requirements, implementation complexity, and cost-benefit trade-offs. This section provides a realistic assessment of deployment challenges and offers scalable implementation strategies for different institutional contexts.

#### Infrastructure requirements and complexity analysis

**Full Implementation Requirements**: The complete DAFF-Net system demands substantial technical infrastructure:

- **Real-time Data Pipeline**: Continuous ingestion from multiple news feeds (Bloomberg, Reuters, PR Newswire), SEC EDGAR filings, and macroeconomic data sources, requiring robust data streaming architecture and 24/7 monitoring
- **Natural Language Processing Infrastructure**: High-performance GPU clusters for real-time BERT encoding of news articles and announcements, with typical latency requirements under 100ms for trading applications
- **Knowledge Graph Maintenance**: Quarterly updates requiring domain expertise to verify corporate relationships, supply chain changes, and regulatory reclassifications from multiple authoritative sources
- **Computational Resources**: Training infrastructure requiring NVIDIA A100-class GPUs and substantial memory for handling large-scale multimodal datasets

**Estimated Implementation Costs**:

- Initial setup: $\$200\text {K}-500\text {K}$ for infrastructure and data subscriptions
- Annual operational costs: $\$150\text {K}-300\text {K}$ for data feeds, cloud computing, and maintenance
- Personnel requirements: 2–3 FTE including ML engineers, data engineers, and domain experts

#### Scalable implementation alternatives

Recognizing that full implementation may be prohibitive for smaller institutions, we propose several graceful degradation strategies that preserve significant portions of DAFF-Net’s performance benefits:

**Tier 1: Simplified Event Integration**

- **Data Sources**: Limit events to earnings announcements, major news headlines, and Fed policy statements
- **Processing**: Use pre-trained sentence transformers instead of full BERT encoding
- **Update Frequency**: Daily batch processing instead of real-time streaming
- **Performance Impact**: Retains approximately 80% of full model improvement
- **Cost Reduction**: 60–70% lower than full implementation

**Tier 2: Relationship-Only Implementation**

- **Focus**: Implement only the multi-dimensional relationship module
- **Data Sources**: Use publicly available fundamental data and static knowledge graphs
- **Event Component**: Replace with simple sentiment scores from free news APIs
- **Performance Impact**: Retains approximately 60% of full model improvement
- **Cost Reduction**: 80% lower than full implementation

**Tier 3: Academic/Research Implementation**

- **Data Sources**: Historical data only, using archived news and fundamental databases
- **Processing**: Offline batch processing suitable for research and backtesting
- **Knowledge Graph**: Simplified using Wikipedia/DBpedia relationships
- **Performance Impact**: Full research validation capability
- **Cost**: Under $\$10\text {K}$ annually for academic research

#### Deployment workflow and best practices

**Phased Implementation Strategy**:

1. **Phase 1 (Months 1–3)**: Implement Tier 3 version for backtesting and validation on institutional data
2. **Phase 2 (Months 4–6)**: Upgrade to Tier 2 with live fundamental data and basic event processing
3. **Phase 3 (Months 7–12)**: Full Tier 1 implementation with comprehensive event integration
4. **Phase 4 (Year 2+)**: Optimization and customization for specific use cases

**Risk Mitigation Strategies**:

- **Fallback Mechanisms**: Maintain traditional LSTM/Transformer models as backup predictors
- **Data Quality Monitoring**: Implement automated data quality checks and anomaly detection
- **Model Validation**: Continuous out-of-sample testing and performance monitoring
- **Regulatory Compliance**: Ensure all data sources and model decisions meet financial regulatory requirements

#### Cost-benefit analysis for different institution types

**Large Asset Managers (**$\${\textbf {10B}}+ {\textbf {AUM}}$**)**: Full implementation justified by potential alpha generation exceeding infrastructure costs. Conservative estimate of 0.5–1.5% annual alpha improvement can generate $\$50-100$M additional returns, easily justifying $\$500$K annual costs.

**Medium Asset Managers (**$\${\textbf {1-10B AUM}}$**)**: Tier 1 implementation recommended. Estimated 0.3–0.7.3.7% alpha improvement generating $\$3-70$M additional returns versus $\$200$K annual costs provides strong ROI.

**Small Asset Managers (**$\${\textbf {100M-1B AUM}}$**)**: Tier 2 implementation most appropriate. Even 0.2–0.4.2.4% alpha improvement generates $\$200$K-4M additional returns versus $\$100$K costs, maintaining positive ROI.

**Individual/Retail Investors**: Tier 3 implementation suitable for educational purposes and personal portfolio management, with minimal costs and research-focused benefits.

#### Technology transfer and open source considerations

To facilitate broader adoption and academic research, we commit to:

- **Open Source Release**: Core model architecture and Tier 3 implementation within 6 months of publication
- **Documentation**: Comprehensive implementation guides and tutorials
- **API Development**: RESTful APIs for simplified integration with existing trading systems
- **Community Support**: GitHub repository with active maintenance and community contributions

This practical implementation framework demonstrates that while DAFF-Net represents a sophisticated advancement in financial prediction, it can be deployed at various scales and resource levels, making its benefits accessible to a broad range of market participants.

## Conclusion

This study proposes the Dual-stream Alpha Factor Fusion Network (DAFF-Net), an innovative deep learning framework specifically designed for financial time series prediction tasks. Through the organic combination of an event-driven temporal pattern extractor and a multi-dimensional relationship-aware channel soft clustering module, DAFF-Net successfully addresses the limitations of traditional financial prediction models in handling complex market narratives and inter-asset correlations. Comprehensive experimental validation on Amazon stock data demonstrates that DAFF-Net significantly outperforms eight representative baseline models including ARIMA, LSTM, Transformer, and DUET across 1-day, 5-day, and 20-day prediction tasks, achieving 7.4%−15.2% improvement in MSE metrics and 7.0%−21.4% enhancement in $\text {R}^{2}$ metrics compared to the strongest baselines, with particularly outstanding advantages in long-term prediction tasks. These results fully demonstrate the effectiveness of deep fusion of event information with multi-dimensional relationship information, providing new technical pathways and theoretical contributions to the field of financial time series prediction. Although current research still has limitations such as limited data coverage and complex technical implementation, DAFF-Net’s core concepts and technical framework open important directions for the development of quantitative investment, risk management, and intelligent financial systems, with broad application prospects and research value. Future work will focus on expanding model validation in multi-asset and cross-market environments, optimizing computational efficiency and deployment convenience, and further enhancing model interpretability to promote the widespread application of event-driven and relationship-aware financial prediction methods in actual financial business.

## Supplementary Information

Supplementary Information.

## Data Availability

The datasets and code used in this study are available upon reasonable request from the corresponding author. Please contact Dr. Jinfei Cao at caojf@szcu.edu.cn for access to the research materials, including the comprehensive multimodal dataset spanning from 2010 to 2025 and the DAFF-Net implementation code.
